# Ethnic minorities treated with new-generation drug-eluting coronary stents in two European randomised clinical trials

**DOI:** 10.1007/s12471-024-01873-9

**Published:** 2024-05-22

**Authors:** Eline H. Ploumen, Edimir Semedo, Carine J. M. Doggen, Carl E. Schotborgh, Rutger L. Anthonio, Peter W. Danse, Edouard Benit, Adel Aminian, Martin G. Stoel, Marc Hartmann, K. Gert van Houwelingen, Martijn Scholte, Ariel Roguin, Gerard C. M. Linssen, Paolo Zocca, Clemens von Birgelen

**Affiliations:** 1 Department of Cardiology, Thoraxcentrum Twente, Medisch Spectrum Twente, Enschede, The Netherlands; 2 Health Technology and Services Research, Faculty of Behavioural, Management and Social Sciences, Technical Medical Centre, University of Twente, Enschede, The Netherlands; 3 Department of Cardiology, Haga Hospital, The Hague, The Netherlands; 4 Department of Cardiology, Treant Zorggroep, Scheper Hospital, Emmen, The Netherlands; 5 Department of Cardiology, Rijnstate Hospital, Arnhem, The Netherlands; 6 Department of Cardiology, Jessa Hospital, Hasselt, Belgium; 7 Department of Cardiology, Centre Hospitalier Universitaire de Charleroi, Charleroi, Belgium; 8 Department of Cardiology, Albert Schweitzer Hospital, Dordrecht, The Netherlands; 9 Department of Cardiology, Hillel Yaffe Medical Centre, Hadera and B. Rappaport-Faculty of Medicine, Israel, Institute of Technology, Haifa, Israel; 10 Department of Cardiology, Hospital Group Twente, Almelo, The Netherlands

**Keywords:** Ethnic minority, Race, Randomised clinical trial, Coronary artery disease, Percutaneous coronary intervention, Drug-eluting stent

## Abstract

**Background:**

Several ethnic minorities have an increased risk of cardiovascular events, but previous European trials that investigated clinical outcome after coronary stenting did not assess the patients’ ethnic background.

**Aims:**

To compare ethnic minority and Western European trial participants in terms of both cardiovascular risk profile and 1‑year clinical outcome after percutaneous coronary intervention.

**Methods:**

In the BIO-RESORT and BIONYX randomised trials*,* which assessed new-generation drug-eluting stents, information on patients’ self-reported ethnic background was prospectively collected. Pooled patient-level data of 5803 patients, enrolled in the Netherlands and Belgium, were analysed in this prespecified analysis. The main endpoint was target vessel failure after 1 year.

**Results:**

Patients were classified as belonging to an ethnic minority (*n* = 293, 5%) or of Western European origin (*n* = 5510, 95%). Follow-up data were available in 5772 of 5803 (99.5%) patients. Ethnic minority patients were younger, less often female, more often current smokers, more often medically treated for diabetes, and more often had a positive family history of coronary artery disease. The main endpoint target vessel failure did not differ between ethnic minority and Western European patients (3.5% vs 4.9%, hazard ratio 0.71, 95% confidence interval 0.38–1.33; *p* = 0.28). There was also no difference in mortality, myocardial infarction, and repeat revascularisation rates.

**Conclusions:**

Despite the unfavourable cardiovascular risk profile of ethnic minority patients, short-term clinical outcome after treatment with contemporary drug-eluting stents was highly similar to that in Western European patients. Further efforts should be made to ensure the enrolment of more ethnic minority patients in future coronary stent trials.

**Supplementary Information:**

The online version of this article (10.1007/s12471-024-01873-9) contains supplementary material, which is available to authorized users.

## Background

Global migration trends have prompted heightened consideration towards the health and medical care of ethnic minorities in various regions of the world. Disparities in health status have been identified among ethnic groups, emphasising the need to optimise risk factor management and medical treatment, tailored to specific subpopulations. Consequently, research involving substantial ethnic minority representation [1, 2] is imperative in order to thoroughly evaluate the efficacy of medical care in routine clinical practice.

While the United States has a tradition of reporting trial results for subgroups of patients with different ethnic backgrounds [3–5], most previous European cardiovascular clinical studies failed to report outcomes related to ethnic background. European studies—assessed until 2018—reported such data in < 5% [6], including clinical trials that investigated the treatment of obstructive coronary artery disease with percutaneous coronary intervention (PCI) using drug-eluting stents (DES). Consequently, in Europe, potential differences in PCI outcome of ethnic minority patients remain largely unknown. This remains an important knowledge gap, given that many ethnic minorities are known to have an elevated risk of rapid progression of coronary artery disease, subsequent hospitalisation, and finally cardiovascular death [7, 8].

**Fig. 1:**
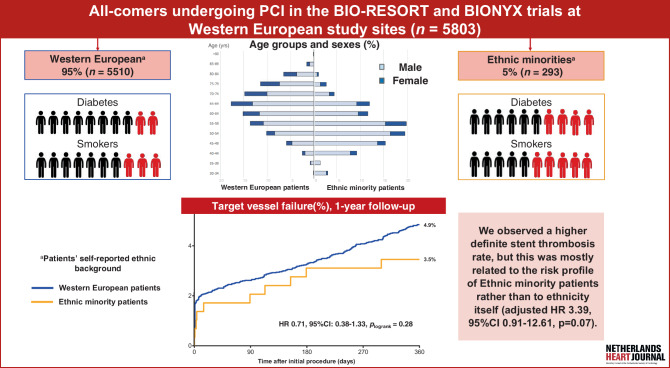
Infographic: Ethnic minority patients were younger, less often female, and more often current smokers. They also more often had medically treated diabetes. Despite their unfavourable cardiovascular risk profile, their short-term outcome was highly similar to that of Western European patients. *CI* confidence interval, *HR* hazard ratio, *PCI* percutaneous coronary intervention

The BIO-RESORT and BIONYX trials [9, 10], two large-scale randomised multicentre studies that evaluated the safety and efficacy of new-generation DES in broad populations of ‘all-comer patients’, prospectively collected information on the self-reported ethnic background of trial participants. We assessed pooled patient-level data from seven study centres in the Netherlands and Belgium and compared ethnic minority patients with Western European patients in terms of cardiovascular risk profile and 1‑year clinical outcome (Fig. 1). The aim of the study was to evaluate whether ethnic minority trial participants have an elevated cardiovascular risk profile that might translate into unfavourable clinical outcome.

## Methods

### Study design and participants

**Fig. 2:**
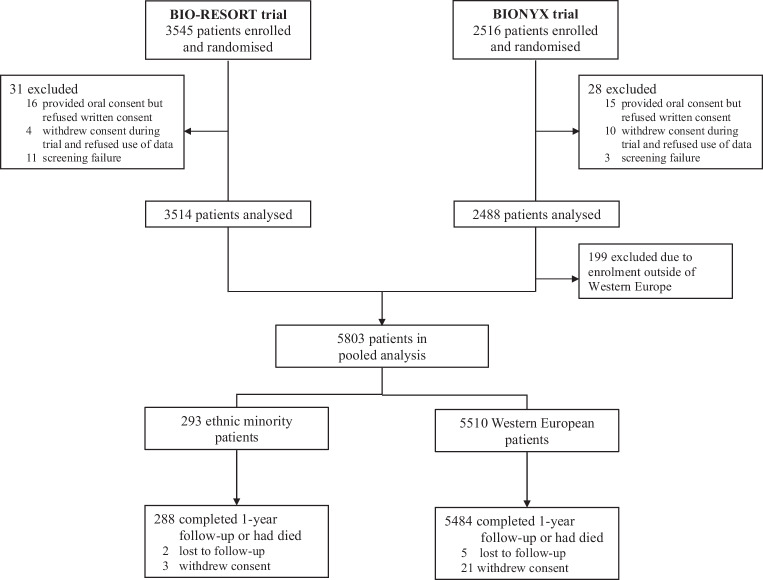
The study flow diagram displays patient enrolled in the two clinical trials and how many patients were included in the current analysis

The study design and details of the BIO-RESORT (*NCT01674803*) and BIONYX (*NCT02508714*) trials have been reported previously [9, 10]. BIO-RESORT was performed in four Dutch centres [9], and BIONYX in four Dutch centres, two Belgian centres, and one centre in Israel [10]. Due to the research question addressed, the current study was restricted to data from Western European centres (Fig. 2). The trials complied with the Declaration of Helsinki and were approved by the Medical Ethics Committee Twente and the institutional review boards of all participating centres. All patients provided written informed consent. Procedures and details of follow-up and monitoring are described in the Electronic Supplementary Material.

### Definitions and clinical endpoints

Prior to randomisation, patients were invited to report their ethnic background. This self-reported information was entered into the database and only accessible to the core study team as part of a coded data set. We report subgroup analyses that were prespecified in both individual study protocols. Western European patients were defined as patients with a self-reported Western European ethnic background. Ethnic minority patients were defined as patients with a self-reported non-Western European ethnic background, such as Eastern or Southern European, Asian, African, or Caribbean (Tab. 1).

**Table 1:** Ethnic background of 5803 study patients who had a percutaneous coronary intervention

	Number of patients (*n* = 5803)
Western European	5510 (95.0)
Ethnic minorities	293 (5.0)
– European, non-Western	32 (0.6)
– Turkish	46 (0.8)
– Asian	84 (1.4)
– Caribbean and Surinam	81 (1.4)
– Moroccan	24 (0.4)
– Other minorities	26 (0.4)

Numbers are *n* (%). Ethnic background was self-reported by study patients. Ethnic minority patients were defined as patients with a self-reported non-native Western European ethnic background. Western European patients were defined as study patients with a Western European ethnic background: e.g. the Netherlands, Belgium, Germany, France, Luxembourg, Switzerland, and Austria. Non-Western European patients were patients from all other European countries, with most patients having a Southern or Eastern European ethnic background

Clinical endpoints were prespecified and in accordance with common definitions [11, 12]. The main composite endpoint was target vessel failure (TVF): cardiac death, target-vessel-related myocardial infarction (MI), or target vessel revascularisation. Secondary endpoints are described in the Electronic Supplementary Material.

### Statistical analysis

Categorical variables were compared using Pearson’s χ^2^ or Fisher’s exact test, as appropriate, and continuous variables using a *t*-test. Time to endpoints was assessed with Kaplan-Meier methods, and a log-rank test was applied for between-group comparisons. Hazard ratios (HR) with two-sided confidence intervals (CI) were computed by Cox proportional hazards analysis. A multivariate analysis considered variables with significant between-group differences (*p* < 0.15 in univariate analysis) and variables expected to be confounders based on the literature (body mass index, sex, smoking), using stepwise backward selection for variable exclusion. The final multivariate model included: age, renal insufficiency, diabetes, previous coronary artery bypass grafting, multi-vessel treatment, and graft treatment. *p*-values < 0.05 were considered significant. Analyses were performed with SPSS version 27 (IBM Corporation, Armonk, NY, USA).

## Results

### Baseline patient characteristics

Of all 5803 trial participants, 293 (5%) were classified as ethnic minority patients and 5510 (95%) as Western European patients. Ethnic minority patients were younger (55.3 vs 64.5 years; *p* < 0.001), less often female (18.4% vs 26.9%; *p* < 0.001), and more often current smokers (46.1% vs 29.3%; *p* < 0.001; Table S2, Electronic Supplementary Material). They more often had medically treated diabetes (27.2% vs 17.5%; *p* < 0.001) and a family history of coronary artery disease (51.4% vs 45.0%; *p* = 0.03). There was no significant between-group difference in the rate of multivessel treatment and lesion complexity (Table S2, Electronic Supplementary Material).

### Clinical outcome

**Fig. 3:**
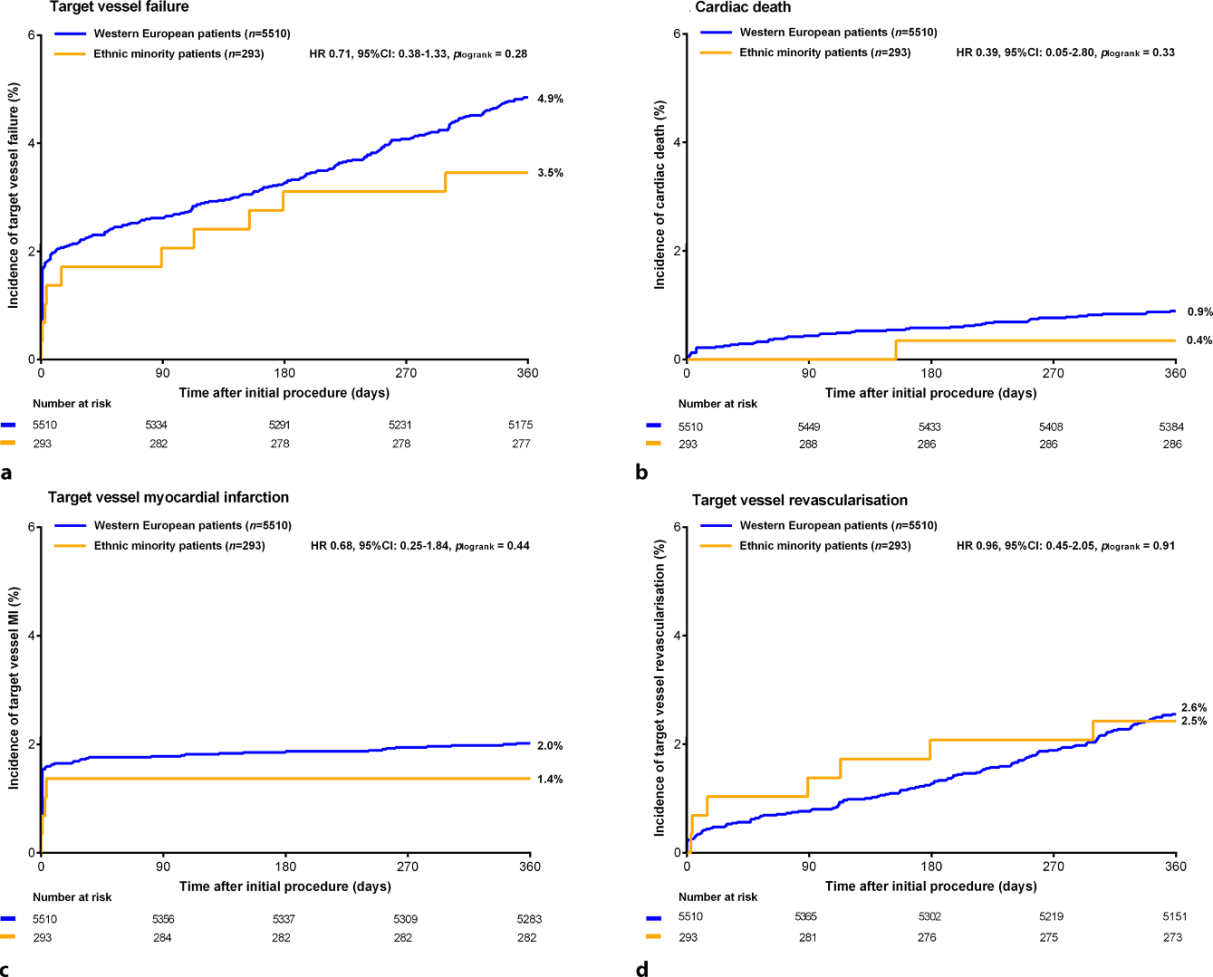
Kaplan-Meier event curves showing the 1‑year clinical outcome of Ethnic Minority and Western European patients. There was no difference in target vessel failure (**a**), cardiac death (**b**), target vessel myocardial infarction (**c**), and target vessel revascularisation (**d**). *CI* confidence interval, *HR* hazard ratio, *MI* myocardial infarction

Follow-up data were available in 5772 (99.5%) patients. Five of 293 (1.7%) ethnic minority patients and 26 of 5510 (0.5%) Western European patients were either lost to follow-up or withdrew their consent (HR: 3.63, 95% CI:1.39–9.44; *p* = 0.005). There was no significant difference in the main endpoint TVF between ethnic minority and Western European patients (3.5% vs 4.9%, HR:0.71, 95% CI:0.38–1.33; *p*_log-rank_ = 0.28, Tab. 2). There was also no between-group difference in its individual components cardiac mortality, target vessel revascularisation, and target vessel MI (Fig. 3; Tab. 2). The incidence of definite stent thrombosis was significantly higher in the ethnic minority group (1.0% vs 0.3%, HR: 3.56, 95% CI: 1.04–12.21; *p*_log-rank_ = 0.03). Composite endpoints target lesion failure and major adverse cardiovascular events (MACE) showed no statistically significant difference (Tab. 2). Use of dual antiplatelet therapy or oral anticoagulants differed between groups at 1‑year follow-up: ethnic minority patients more often used dual antiplatelet therapy but less often used oral anticoagulants (Table S1, Electronic Supplementary Material).

**Table 2:** Clinical outcomes

	Ethnic minority patients (*n* = 293)	Western European patients (*n* = 5510)	*P*_log-rank_	HR (95% CI)	*P*_log-rank_	Adjusted HR^a^ (95% CI)
Target vessel failure	10 (3.5)	266 (4.9)	0.28	0.71 (0.38–1.33)	0.65	0.86 (0.45–1.64)
Any death	2 (0.7)	102 (1.9)	0.15	0.37 (0.09–1.50)	0.46	0.59 (0.14–2.41)
– Cardiac death	1 (0.4)	49 (0.9)	0.33	0.39 (0.05–2.80)	0.73	0.70 (0.9–5.22)
Any MI	5 (1.7)	117 (2.1)	0.63	0.80 (0.33–1.97)	0.96	1.02 (0.41–2.55)
– Target vessel MI	4 (1.4)	111 (2.0)	0.44	0.68 (0.25–1.84)	0.78	0.87 (0.32–2.39)
Any revascularisation	13 (4.5)	224 (4.5)	0.96	1.01 (0.58–1.77)	0.88	0.96 (0.54–1.69)
– Target vessel revascularisation	7 (2.5)	139 (2.6)	0.91	0.96 (0.45–2.05)	0.95	0.98 (0.45–2.12)
– Target lesion revascularisation	4 (1.4)	97 (1.8)	0.63	0.78 (0.29–2.12)	0.75	0.85 (0.31–2.35)
– Non-target vessel revascularisation	6 (2.1)	105 (1.9)	0.85	1.08 (0.48–2.47)	0.86	0.93 (0.40–2.15)
Target lesion failure	7 (2.4)	227 (4.1)	0.15	0.58 (0.27–1.23)	0.46	0.75 (0.35–1.61)
MACE	9 (3.1)	281 (5.1)	0.13	0.60 (0.31–1.17)	0.48	0.79 (0.40–1.54)
POCE	17 (5.9)	414 (7.5)	0.30	0.78 (0.48–1.26)	0.57	0.87 (0.53–1.42)
Definite or probable stent thrombosis	3 (1.0)	23 (0.4)	0.13	2.48 (0.74–8.25)	0.25	2.09 (0.59–7.44)
– Definite stent thrombosis	3 (1.0)	16 (0.3)	0.03	3.56 (1.04–12.21)	0.07	3.39 (0.91–12.61)

Numbers are *n* (%) representing 1‑year clinical outcomes

*CI* confidence interval, *HR* hazard ratio, *MACE* major adverse cardiac events, *MI* myocardial infarction, *POCE* patient-oriented composite endpoint

^a^ Adjusted HR: adjustments were made for confounders for the main composite endpoint TVF, i.e. age, renal insufficiency, diabetes, previous coronary artery bypass grafting, multivessel treatment, and graft treatment

After adjustment for potential confounders, ethnic minority background was not independently associated with TVF (adjusted HR: 0.86, 95% CI: 0.45–1.64; *p*_log-rank_ = 0.65) or other clinical outcomes (Tab. 2). In particular, ethnic minority background was no longer significantly associated with definite stent thrombosis (adjusted HR: 3.39, 95% CI: 0.91–12.61; *p*_log-rank_ = 0.07).

## Discussion

Ethnic minority patients represented 5% of the overall 5803 patients. They differed in cardiovascular risk profile from Western European patients, and although ethnic minority patients were much younger (55 vs 65 years), they had a higher prevalence of diabetes (27% vs 18%). Furthermore, they were less often female and more often current smokers. Treatment of multivessel disease was performed in 14% of ethnic minority and 19% of Western European patients. While this may suggest a somewhat lower disease burden in the trial participants with an ethnic minority background, the rate of patients with complex (i.e. calcified or bifurcated) target lesions did not differ between groups. The 1‑year rate of the main endpoint TVF did not differ between groups (3.5% vs 4.9%), and multivariate analysis with adjustment for confounders did not change this finding. In addition, there was no between-group difference in mortality, MI, and repeated revascularisation rates. While ethnic minority patients had a higher definite stent thrombosis rate (1.0% vs 0.3%), multivariate analysis revealed that ethnic background was not independently associated with stent thrombosis.

### Cardiovascular risk profile of ethnic minority patients

Earlier studies, assessing health disparities in countries with a Western lifestyle, have shown that ethnic minorities have an increased burden of cardiovascular risk factors such as diabetes and hypertension [13, 14]. The present study demonstrates that ethnic minority patients presenting with obstructive coronary artery disease were on average younger and had more risk factors, including diabetes, smoking, and a family history of coronary disease. This is in line with previous reports [15–17]. Our analysis shows that after adjustment for these potential confounding factors, in ethnic minority patients the event risk of our main clinical endpoint TVF was even lower (HR closer to 1.0). This suggests that not the ethnic background but the patient’s risk profile determines the incidence of adverse events. Despite differences in cardiovascular risk profile, 1‑year clinical outcome was similar in ethnic minority and Western European patients. Nevertheless, intensifying secondary prevention measures will be of great importance in preventing adverse events in the long term [18], as patients of various ethnic backgrounds can benefit from optimising cardiovascular risk profiles [19].

### Clinical outcome of ethnic minority patients with different stents

Most previous studies that assessed ethnical background in the context of coronary stenting were conducted in the eras of bare metal stents and first-generation DES. These studies suggested an increased adverse event risk in ethnic minority patients as compared to non-immigrants [7, 13]. Meanwhile, there has been substantial progress in DES design, PCI techniques, and concomitant medication [20, 21].

The present study in ethnic minority patients treated with new-generation DES did not show an elevated event risk, which is in line with a recent study from the United States [15]. That study pooled 4182 patients, treated with new-generation DES, from two observational studies and found no impact of ethnic background on 1‑year risks of MACE, mortality, and target vessel revascularisation. Yet, when the individual components of MACE were separately assessed, ethnic minorities encountered a higher risk of recurrent MI, primarily driven by non-stent-related MI [15]. This may reflect their less favourable cardiovascular risk profile. In addition, differences in health politics (e.g. general access to healthcare) may also account for a higher risk in patients with low socioeconomic status from the United States, who may have difficulties with medication adherence due to financial restrictions. In the present study, we did not encounter a higher risk of recurrent MI in the ethnic minority group.

Another analysis of pooled data from two observational studies, conducted in Europe and Asia, assessed potential differences between ethnic groups in risk profile and clinical outcome after PCI with polymer-free drug-coated stents [22]. The event rates in that study were similar to the event rates in our analysis with contemporary DES. Another study that evaluated use of a biodegradable polymer DES with anti-CD34+ antibody coating in Asian and European patients found a higher incidence of diabetes in Asian patients, similar to our ethnic minority population. In addition, our study findings are in line with their results that showed no short-term difference in adjusted clinical outcomes between Asian and European patients [23]. Furthermore, a nationwide cohort study in the United Kingdom assessed ethnic disparities in care and clinical outcome of patients who were treated with PCI or coronary artery bypass grafting for non-ST-segment elevation MI [17]. Ethnic minorities more often underwent repeated revascularisation, but they had rates of in-hospital mortality, MACE, and major bleeding that were similar to those of White patients.

### Stent thrombosis

In the present study, the ethnic minority patients had a higher incidence of definite stent thrombosis, but there was no independent association with ethnic background. This finding is in contrast to earlier studies from the United States, which found that ethnic (particularly African American) background was an independent risk factor for thrombus formation in first-generation DES [24]. Others explained inferior outcome in ethnic minority patients by different stent choices and the fact that African Americans were more likely to be treated with bare metal stents than White patients [25, 26]. Yet, the stent choice alone is unlikely to explain a higher rate of stent thrombosis.

In current clinical practice in the Netherlands and Belgium, discriminating stent use is highly unlikely, as DES implantation is the standard of care and new-generation DES are widely available [20, 21]. Likewise, in our present study, trial participants of all ethnic backgrounds were treated with randomly allocated new-generation DES, which precludes any stent choice. We can only speculate that our observation of a higher definite stent thrombosis rate in the unadjusted analysis is likely related to the higher cardiovascular risk profile that we observed in ethnic minority patients. The fact that the association between stent thrombosis and ethnic minority status was no longer significant after multivariate adjustment supports this notion. However, the overall incidence of stent thrombosis was low, and these results should be interpreted with caution.

### Limitations

All findings should be considered hypothesis generating. As the number of patients with an ethnic minority background was modest, we pooled data from two similar trials. Most randomised trials fail to represent the full spectrum of patients with an ethnic minority background treated in routine clinical practice. Patients unable to (adequately) speak the local language, required for informed consent, are generally not considered for randomised trials, resulting in enrolment of fewer ethnic minority patients; this also applies to the present study. Data regarding self-reported ethnicity of non-enrolled patients was not available, so it is not known how many ethnic minority patients did not participate. The possible language barrier could also lead to enrolment of younger patients in the ethnic minority group, as they are generally more proficient in the local language. Although we adjusted for age in the multivariate model, this could potentially still impact clinical outcomes. Health disparities between ethnic minority and Western European patients encompass not only cardiovascular risk profiles, but also social determinants of health [27, 28], which are difficult to measure and were not documented in this study. A particular challenge in studying ethnic disparities is the heterogeneity and genetic diversity within ethnic minority groups. Given the small size of most ethnic subgroups, we chose a straightforward approach that categorised trial participants as ethnic minority or Western European patients. Yet, we acknowledge that allocating all patients with an ethnic minority background to a single group combines ethnic subgroups with varying cardiovascular risk profiles and social health determinants. Nevertheless, we felt that the chosen pragmatic approach was sound and perhaps the only way to obtain meaningful results.

## Conclusions

Despite the unfavourable cardiovascular risk profile of the ethnic minority trial participants, 1‑year clinical outcome after treatment with contemporary drug-eluting stents was highly similar to that of Western European patients. After adjustment for dissimilarities in baseline cardiovascular risk factors, the adverse event risk of both groups was even more similar, suggesting that the cardiovascular risk profile—rather than the ethnic background itself—determines the event risk. Further efforts should be made to ensure the enrolment of more ethnic minority patients in future coronary stent trials.

### Supplementary Information

Additional Methods and Results

Table S2, see attachment.
