# Supplementary material for: Ethnic minorities treated with new-generation drug-eluting coronary stents in two European randomised clinical trials
Source: Neth Heart J. 2024 May 22;32(6):254–61. doi: 10.1007/s12471-024-01873-9 (PMC11143136; doi:10.1007/s12471-024-01873-9)
Supplement: Supplementary file 1 — Additional Methods and Results [file 12471_2024_1873_MOESM1_ESM.pdf]

# SUPPLEMENTAL MATERIAL

## Ethnic minorities treated with new-generation drug-eluting coronary stents in two European randomized clinical trials

Eline H. Ploumen, MD PhD,[1,2] Edimir Semedo, MD,[1,2] Carine J.M. Doggen, PhD,[2]
Carl E. Schotborgh, MD,[3] Rutger L. Anthonio MD PhD,[4] Peter W. Danse, MD PhD,[5]
Edouard Benit, MD,[6] Adel Aminian, MD,[7] Martin G. Stoel, MD PhD,[1] Marc Hartmann, MD PhD,[1]
K. Gert van Houwelingen, MD,[1] Martijn Scholte, MD,[8] Ariel Roguin, MD PhD,[9]
Gerard C.M. Linssen, MD PhD,[10] Paolo Zocca, MD PhD,[1] Clemens von Birgelen, MD PhD,[1,2]

1 Department of Cardiology, Thoraxcentrum Twente, Medisch Spectrum Twente, Enschede, the Netherlands
2 Health Technology and Services Research, Faculty of Behavioural, Management and Social Sciences, Technical Medical Centre, University of Twente, Enschede, the Netherlands
3 Department of Cardiology, Haga Hospital, The Hague, the Netherlands
4 Department of Cardiology, Treant Zorggroep, Scheper Hospital, Emmen, the Netherlands
5 Department of Cardiology, Rijnstate Hospital, Arnhem, the Netherlands
6 Department of Cardiology, Jessa Hospital, Hasselt, Belgium
7 Department of Cardiology, Centre Hospitalier Universitaire de Charleroi, Charleroi, Belgium
8 Department of Cardiology, Albert Schweitzer Hospital, Dordrecht, the Netherlands
9 Department of Cardiology, Hillel Yaffe Medical Center, Hadera and B. Rappaport-Faculty of Medicine, Israel, Institute of Technology, Haifa, Israel
10 Department of Cardiology, Hospital Group Twente, Almelo and Hengelo, the Netherlands

## Supplemental Methods

### Procedures, follow-up, and monitoring

Patients were eligible for enrolment in the main trials if they were 18 years or older, capable of providing informed consent, and required percutaneous coronary intervention. There was no restriction for target lesion type or length, reference vessel size, number of vessels or lesions, and clinical syndrome at presentation.

In the BIO-RESORT trial, patients were randomized to treatment with sirolimus-eluting Orsiro (Biotronik, Bülach, Switzerland), everolimus-eluting Synergy (Boston Scientific, Marlborough, MA), or zotarolimus-eluting Resolute Integrity stents (Medtronic, Santa Rosa, CA).[9] In the BIONYX trial, trial participants were randomized to treatment with zotarolimus-eluting Resolute Onyx (Medtronic) or Orsiro stents.[10]

Coronary interventions and the choice of concomitant medication were based on routine clinical practice, current guidelines, and operator's judgement. Trial and data management was performed by Cardiovascular Research and Education Enschede (Enschede, the Netherlands). Clinical follow-up was obtained at visits to outpatient clinics, by telephone, or by questionnaire. Data monitoring and clinical event adjudication were performed by independent external research organizations.

### Secondary endpoints

Secondary endpoints included the individual components of target vessel failure, target lesion revascularization, and stent thrombosis. Other secondary composite endpoints were target lesion failure (cardiac death, target vessel myocardial infarction, or target lesion revascularization), major adverse cardiac events (all-cause death, any myocardial infarction, or target lesion revascularization), and patient oriented composite endpoint (all-cause death, any myocardial infarction, or any revascularization).

## Supplemental Results

**Supplemental Table S1. Use of antiplatelet therapy and oral anticoagulants at 1-year follow-up**

| | **Ethnic Minority Patients** (n=293) | **Western European Patients** (n=5,510) | $P_{log-rank}$ |
|---|---|---|---|
| **Acetylsalicylic acid** | 275/286 (96.2%) | 4,698/5,420 (86.7%) | <0.001 |
| **Dual antiplatelet therapy** | 269/286 (94.1%) | 4,557/5,382 (84.7%) | <0.001 |
| **Clopidogrel** | 119/286 (41.6%) | 2,699/5,382 (50.1%) | 0.005 |
| **Ticagrelor** | 149/286 (52.1%) | 2,332/5,382 (43.3%) | 0.004 |
| **Prasugrel** | 12/287 (4.2%) | 158/5434 (2.9%) | 0.22 |
| **Oral anticoagulant** | 9/285 (3.2%) | 625/5,328 (11.7%) | <0.001 |
