# Supplementary material for: Ethnic minorities treated with new-generation drug-eluting coronary stents in two European randomised clinical trials
Source: Neth Heart J. 2024 May 22;32(6):254–61. doi: 10.1007/s12471-024-01873-9 (PMC11143136; doi:10.1007/s12471-024-01873-9)
Supplement: Supplementary file 2 — Table S2, see attachment. [file 12471_2024_1873_MOESM2_ESM.docx]

**Table 2** Baseline patient and lesion characteristics

|  | **Ethnic**  **minority patients**  (*n*=293) | **Western European patients**  (*n*=5510) | ***p*** |
| --- | --- | --- | --- |
| Age (years) | 55.3 (±10.2) | 64.5 (±10.7) | <0.001 |
| Female | 54 (18.4) | 1480 (26.9) | 0.001 |
| BMI (kg/m^2^) | 27.5 (±4.3) | 27.6 (±4.3) | 0.65 |
| Diabetes mellitus | 80 (27.3) | 966 (17.5) | <0.001 |
| Arterial hypertension | 129 (44.0) | 2622 (47.9) | 0.20 |
| Hypercholesterolaemia | 106 (36.2) | 2194 (40.3) | 0.16 |
| Current smoker | 135 (46.1) | 1568 (29.3) | <0.001 |
| Family history of CAD | 148 (51.6) | 2373 (45.0) | 0.03 |
| Chronic renal insufficiency^a^ | 8 (2.7) | 256 (4.6) | 0.13 |
| Previous myocardial infarction | 54 (18.4) | 952 (17.3) | 0.61 |
| Previous PCI | 47 (16.0) | 1039 (18.9) | 0.23 |
| Previous CABG | 14 (4.8) | 414 (7.5) | 0.08 |
| Left ventricular ejection fraction <30% | 2 (0.7) | 85 (1.5) | 0.24 |
| Clinical presentation at admission |  |  | 0.05 |
| Acute coronary syndrome | 221 (75.4) | 3864 (70.1) |  |
| Stable angina | 72 (24.6) | 1646 (29.9) |  |
| Multivessel treatment | 41 (14.0) | 1017 (18.5) | 0.05 |
| Left main treated | 4 (1.4) | 115 (2.1) | 0.40 |
| LAD treated | 156 (53.2) | 2705 (49.1) | 0.17 |
| RCA treated | 103 (35.2) | 2099 (38.1) | 0.31 |
| RCX treated | 72 (24.6) | 1598 (29.0) | 0.10 |
| Graft treated | 2 (0.7) | 105 (1.9) | 0.13 |
| At least one bifurcation treated | 116 (39.6) | 2021 (36.7) | 0.31 |
| At least one severely calcified lesion | 64 (22.1) | 1122 (20.3) | 0.54 |
| At least one ACC/AHA complex lesion type (B2 and C) | 228 (77.8) | 4308 (78.2) | 0.88 |
| Stent type |  |  | 0.78 |
| Orsiro | 112 (38.2) | 2205 (40.0) |  |
| Synergy | 63 (21.5) | 1109 (20.1) |  |
| Resolute Onyx | 52 (17.7) | 1089 (19.8) |  |
| Resolute Integrity | 66 (22.5) | 1107 (20.1) |  |

Numbers are *n* (%) or mean (±SD)

^a^Chronic renal insufficiency was defined as an estimated glomerular filtration rate of less than 30 ml/min per 1.73 m² or need for dialysis

*ACC/AHA* American College of Cardiology/American Heart Association, *BMI* body mass index, *CAD* coronary artery disease, *CABG* coronary artery bypass graft, *LAD* left anterior descending artery, *PCI* percutaneous coronary intervention, *RCA* right coronary artery, *RCX* ramus circumflexus
